# A Novel Method for Asynchronous Time-of-Arrival-Based Source Localization: Algorithms, Performance and Complexity

**DOI:** 10.3390/s20123466

**Published:** 2020-06-19

**Authors:** Yuanpeng Chen, Zhiqiang Yao, Zheng Peng

**Affiliations:** 1 Intelligent Navigation and Remote Sensing Research Center, Xiangtan University, Xiangtan 411105, China; lesliechan0513@gmail.com; 2 Changsha Technology Research Institute of Beidou Industry Safety, Changsha 410006, China; 3 School of Mathematics and Computational Science, Xiangtan University, Xiangtan 411105, China; pzheng@xtu.edu.cn

**Keywords:** source localization, time-of-arrival, signal processing, asynchronous sensor networks

## Abstract

In time-of-arrival (TOA)-based source localization, accurate positioning can be achieved only when the correct signal propagation time between the source and the sensors is obtained. In practice, a clock error usually exists between the nodes causing the source and sensors to often be in an asynchronous state. This leads to the asynchronous source localization problem which is then formulated to a least square problem with nonconvex and nonsmooth objective function. The state-of-the-art algorithms need to relax the original problem to convex programming, such as semidefinite programming (SDP), which results in performance loss. In this paper, unlike the existing approaches, we propose a proximal alternating minimization positioning (PAMP) method, which minimizes the original function without relaxation. Utilizing the biconvex property of original asynchronous problem, the method divides it into two subproblems: the clock offset subproblem and the synchronous source localization subproblem. For the former we derive a global solution, whereas the later is solved by a proposed efficient subgradient algorithm extended from the simulated annealing-based Barzilai–Borwein algorithm. The proposed method obtains preferable localization performance with lower computational complexity. The convergence of our method in Lyapunov framework is also established. Simulation results demonstrate that the performance of PAMP method can be close to the optimality benchmark of Cramér–Rao Lower Bound.

## 1. Introduction

In the Wireless Sensor Networks (WSNs), the concept of wireless interconnection between sensors brings hope for breakthrough in new application fields [1]. In most application scenarios, e.g., battlefield surveillance, forest fire detection, managing inventory control and smart homes, the location information of sensors can be obtained via Global Positioning System (GPS) or source localization. In source localization, the sensors with known position measure the signals transmitted from a source, and then relay the noisy measurement data to a signal processing center for further processing and data fusion. Finally, the center estimates the location of source based on the source localization algorithm [2]. In terms of algorithms, So summarized some basic source localization approaches from linear and nonlinear aspects [3].

Currently, the source localization algorithms mainly depend on range measurement. It can be extracted from the received radio signal data using the following measurement approaches: received-signal-strength (RSS) [4], angel-of-arrival (AOA) information [5], time-of-arrival (TOA) [6], time-difference-of-arrival (TDOA) [7] and their combination [8]. It is well known that time-based ranging techniques can achieve high localization accuracy at lower power consumption, especially using TOA. The TOA technique requires strict clock synchronization between the source and the sensors [2]. However, in many engineering applications, even though the sensors are synchronized (via GPS timing), there is still an initial clock offset $t_{0}$ between the source and the sensors. The sensors cannot obtain the emitted time of the radio signal sent by the source because of $t_{0}$. In the absence of accurate information on clock offset, the measurement of arrival time will deviate from its true value. Although it seems to be only a tiny error, its influence when speed of light is considered can greatly affect the positioning accuracy. In fact, TOA ranging technique is more reliable than others in underwater acoustic sensor networks (UASNs) [9], and the speed of sound will also affect the positioning accuracy due to the existence of $t_{0}$. Hence, we focus on the asynchronous TOA-based source localization in this paper.

Finding an efficient method for the original problem can solve asynchronous problem fundamentally, which is a big challenge. In this paper, we present a proximal alternating minimization positioning (PAMP) method for directly dealing with the asynchronous TOA-based source localization problem. The proposed method divides the original problem into two subproblems based on the special structure of the objective: the clock offset subproblem and the synchronous source localization subproblem. The former subproblem has a closed-form solution, and the later one is solved by an efficient Barzilai–Borwein algorithm using subgradient information. Then the two subproblems are alternately minimized until the joint estimate is obtained. The main contributions of this paper are as follows:

(1) We propose a proximal alternating minimization positioning (PAMP) method for the original asynchronous TOA-based source localization problem, and the simulated annealing-based Barzilai–Borwein subgradient (SABB-Subgradient) algorithm is extended from the simulated annealing-based Barzilai–Borwein (SABB) algorithm to solve the subproblem.
(2) The convergence and Cramér–Rao performance analysis of the proposed method are presented.
(3) Even in the complex environment with high noise, the algorithm can still meet the requirements of high precision and real-time positioning.
(4) Proposed algorithms do not depend on a specific noise distribution and in the process of solving, these do not need to use the noise structure. For any noise distribution we can always find a superior solution.
(5) As is apparently illustrated by the numerical results, the proposed method has the best performance compared with the existing approaches. In the sense, it has the minimal gap with CRLB. At the same time, the proposed method also has the least computational time.

The rest of the paper is organized as follows. Section 2 provides an overview of existing approaches. Section 3 describes asynchronous TOA-based source localization problem and formulates it to a least square problem. Then the proposed PAMP method and SABB-Subgradient algorithm are described in detail. The convergence analysis is also presented in this section. In Section 4, the performance bound is deduced from Fisher information matrix, and the effectiveness and superiority of the proposed method are illustrated by numerical simulations. The last section summarizes this paper by some final remarks.

## 2. Related Work

There are four ways to deal with the asynchronous TOA-based source localization problem.

The first one is two-way TOA technique [10], it requires that the signal is sent from sensor to source, and then the round trip time between the sensor and the source is measured to solve an asynchronous problem.

The next solution is TDOA measurement [7]. Select an sensor as the reference node, the asynchronous effect is mitigated by subtracting the TOA measurements on the reference node from the TOA measurements of other sensors [2]. The advantage of TDOA technique is that synchronization between the sensors and the source is not required. Yang et al. [7] considered a maximum likelihood formula for the target location problem and provided effective convex relaxation for the resulted non-convex optimization problem. In the case of large measurement noise, the performance of TDOA is significantly better than the existing methods based on the least squares.

The third way is joint source position and time synchronization [11], this kind of TOA measurement model considers clock skew and clock offset at the same time, the positioning accuracy is relatively high. Zheng et al. [11] deduced a maximum likelihood estimator of this model, which has a highly nonlinear, non-convex objective function. As a result that the original function has no closed-form solution, the traditional algorithm consumes too much energy, they proposed a joint maximum likelihood estimation and a two-step least squares estimation with higher computational efficiency.

The last one directly estimates the source location [12,13,14], this type of model only contains clock offset in clock parameters, the simpler model structure leads to lower computational complexity. In [12], Xu et al. proposed a two-step estimation algorithm and a min-max algorithm, both of which are based on semidefinite programming (SDP) technique. In [13], Vaghefia et al. proposed a novel SDP estimator which estimates the source transmit time jointly with the source location. In 2016, Zou et al. [14] used SDP skill to convexify the original problem, in order to improve the tightness of the original SDP algorithms, SOC constraint is combined with an additional penalty term. The usual relaxation techniques of these algorithms are summarized as follows:

(1) Based on least square:

In 2LS approach, the estimation of $t_{0}$ is obtained by using the least squares technique:

(1) $${\hat{t}}_{0}=\frac{1}{N}\sum_{i=1}^{N}\left(t_{i}-\frac{1}{c}||s-a_{i}||\right).$$

Then substitute $t_{0}$ with ${\hat{t}}_{0}$:

(2) $$\sum_{i=1}^{N}{\left[t_{i}-\frac{1}{c}||s-a_{i}||-\frac{1}{N}\sum_{j=1}^{N}\left(t_{j}-\frac{1}{c}||s-a_{j}||\right)\right]}^{2}.$$

The resulting objective function is still nonconvex. After introducing auxiliary variables $\tau_{i}=\frac{1}{c}||s-a_{i}||$, $G=I-\frac{1}{N}1\cdot 1^{T}$, $Q=\tau \tau^{T}$ and $y_{s}=s^{T}s$, the final objective function and constraint of 2LS algorithm can be obtained:

(3) $$\begin{array}{c} \min_{s,y_{s},\tau ,Q}\mathrm{Tr}\left[G^{T}G\left(Q-2t\tau^{T}+tt^{T}\right)\right]+\eta \sum_{i=1}^{N}\sum_{j=1}^{N}Q_{ij}\\ s.t.\left[\begin{array}{cc} Q & \tau \\ \tau^{T} & 1 \end{array}\right]⪰0,\left[\begin{array}{cc} I & s\\ s^{T} & y_{s} \end{array}\right]⪰0,\\ Q_{ii}=\frac{1}{c^{2}}{\left[\begin{array}{c} a_{i}\\ -1 \end{array}\right]}^{T}\left[\begin{array}{cc} I & s\\ s^{T} & y_{s} \end{array}\right]\left[\begin{array}{c} a_{i}\\ -1 \end{array}\right],\\ Q_{ij}\geq \frac{1}{c^{2}}\left|{\left[\begin{array}{c} a_{i}\\ -1 \end{array}\right]}^{T}\left[\begin{array}{cc} I & s\\ s^{T} & y_{s} \end{array}\right]\left[\begin{array}{c} a_{j}\\ -1 \end{array}\right]\right|,\\ i=1,\cdots ,N,j=i+1,\cdots ,N, \end{array}$$

where $\eta \sum_{i=1}^{N}\sum_{j=1}^{N}Q_{ij}$ is the penalty term, which is used for avoiding the ambiguity of original function. Semidefinite relaxation is applied to relax the auxiliary variables into convex inequalities in the constraints.

(2) Based on min-max criterion:

The MMA approach utilizes the idea of minimizing the peak error to construct the cost function as:

(4) $$\begin{array}{c} \hat{s}=\arg\min_{s,y_{s},t_{0},t_{s}}\max_{i=1,\cdots ,N}\left|t_{s}-2t_{i}t_{0}+t_{i}^{2}\right.\left.-\frac{1}{c^{2}}\left(y_{s}-2a_{i}^{T}s+a_{i}^{T}a_{i}\right)\right|, \end{array}$$

where $y_{s}=s^{T}s$ and $t_{s}=t_{0}^{2}$ are auxiliary variables. By the same operation as semidefinite relaxation in 2LS algorithm, the final objective function can be expressed as:

(5) $$\begin{array}{c} \min_{s,y_{s},t_{0},t_{s}}\theta \\ s.t.-\theta \leq t_{s}-2t_{i}t_{0}+t_{i}^{2}-\frac{1}{c^{2}}\left(y_{s}-2a_{i}^{T}s+a_{i}^{T}a_{i}\right)\leq \theta ,\\ i=1,\cdots ,N,\\ \left[\begin{array}{cc} 1 & t_{0}\\ t_{0} & t_{s} \end{array}\right]⪰0,\left[\begin{array}{cc} I & s\\ s^{T} & y_{s} \end{array}\right]⪰0. \end{array}$$

(3) Based on additional Second-Order Cone constraint:

The SDP2016 algorithm utilizes the same two-step LS technique as the 2LS algorithm, with only two differences. The first is that the penalty term in SDP2016 is ηtr(D), which is a more sensitive tuning parameter. The second difference is that the constraint is more tightness by the additional SOC constraint. Define a new vector $V_{i}=\left(d_{i},s-a_{i}\right)\in R^{n+1},i=1,2,\ldots ,N$, where $d_{i}=||X(:,1)-X(:,i+1)||$, $X=[s,a_{1},a_{2},\ldots ,a_{N}]$. Then the SOC constraint can be expressed as $V_{i}\left(1\right)\geq ||V_{i}\left(2:\left(n+1\right)\right)||,i=1,2,\ldots ,N$, i.e., $d_{i}\geq ||s-a_{i}||,i=1,2,\ldots ,N$.

At present, the algorithms for solving asynchronous problems still have the following defects. The two-way TOA technique is not applicable to active localization, i.e., the source node should have the ability to send and receive data, whereas the traditional TOA technique only requires the source node to send data. Although the TDOA technique can eliminate the effect of clock offset by subtraction of pairwise TOA measurements, it also increases the correlation between measurements. These correlated will enhance the measurement noise of 3 dB. In the joint estimation, the variables were lifted into a higher dimensional space, which in turn increases the computational complexity. The last model is simpler than the previous ways, but the existing SDP algorithms with better performance all contain penalty term, which is an uncontrollable and extremely sensitive factor, so it is difficult to configure an appropriate penalty factor to solve the actual asynchronous source localization problem. Their computation time are still high, which is unacceptable for a real-time positioning system.

## 3. Problem Statement and Proposed Algorithms

**Figure 1:**
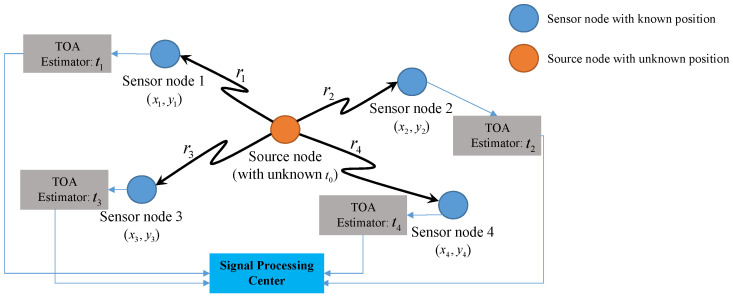
Asynchronous time-of-arrival (TOA)-based source localization.

Suppose, there is a wireless sensor network having *N* distributed sensors with known positions and one source node with unknown position, as shown in Figure 1. Let $a_{i}={\left(x_{i},y_{i}\right)}^{T}\left(i=1,2,\cdots ,N\right)$ and $s={\left(x,y\right)}^{T}$ be the position of the *i*-th sensor and the source node to be located respectively. Then, for the signal propagation along the line of sight (LOS) path, the TOA measurement at the *i*-th sensor is:

(6) $$t_{i}=\frac{1}{c}{∥a_{i}-s∥}_{2}+t_{0}+n_{i},i=1,2,\cdots ,N,$$

where $t_{0}$ is the initial clock offset between the sensors and the source node, *c* is the speed of light or the speed of sound. The term $∥a_{i}{-s∥}_{2}$ is the distance from the *i*-th sensor to the source node, and $n_{i}$ is the measurement noise (error) between the *i*-th sensor and the source that can be arbitrarily distributed. Without any prior assumptions on the statistics of the TOA measurements, the least square (LS) estimation of *s* and $t_{0}$ is as follows:

(7) $$\min_{s,t_{0}}f\left(s,t_{0}\right)=\frac{1}{2}\sum_{i=1}^{N}{\left[c*\left(t_{i}-t_{0}\right)-{\left||a_{i}-s|\right|}_{2}\right]}^{2}.$$

This model is called the range-based least squares (RLS) [15] model.

### 3.1. Proposed Subproblems and Algorithms

Problem (7) is highly non-convex and non-smooth on jointed variable $\left(s,t_{0}\right)$, so it is a challenge for finding a global solution. Fortunately, the objective function is biconvex, i.e., convex on one variable if the other is fixed. This feature motivates the proximal alternating minimization positioning (PAMP) method as follows. For a given $\left(s^{k},t_{0}^{k}\right)$, we find the next iteration $\left(s^{k+1},t_{0}^{k+1}\right)$ via

(8) $$\left\{\begin{array}{cc} t_{0}^{k+1}= & \arg\min_{t_{0}}f\left(s^{k},t_{0}\right)+\frac{r}{2}{\left(t_{0}-t_{0}^{k}\right)}^{2},\\ s^{k+1}= & \arg\min_{s}f\left(s,t_{0}^{k+1}\right). \end{array}\right.$$

The PAMP method divides the complex nonconvex problem (7) into two subproblems: one is clock offset subproblem with respect to $t_{0}$, and the other is synchronous source localization subproblem with respect to *s*. At the *k*-th iteration, two subproblems in (8) are convex. For fixed $s=s^{k}$, the clock offset subproblem with the location $s^{k}$ estimated by the previous step is a smoothing convex minimization problem, which has a closed-form solution, and it is also a global solution. With fixed $t_{0}=t_{0}^{k+1}$ estimated in the current step, the asynchronous source localization problem reduces to a synchronous source localization problem. The source localization problem is nonsmooth but convex with respect to *s*. Hence, the approximating global solution could be obtained by some subgradient minimization algorithms.

#### 3.1.1. Clock Offset Subproblem with Known Location

In clock offset subproblem, the source node location $s^{k}$ estimated in the last step is used as a known location, then the current estimator of clock offset can be expressed as:

(9) $$t_{0}^{k+1}=\arg\min_{t_{0}}\left\{f\left(s^{k},t_{0}\right)=\frac{1}{2}\sum_{i=1}^{N}{\left[c*\left(t_{i}-t_{0}\right)-||a_{i}-s^{k}||\right]}^{2}\right\}.$$

The subproblem is to estimate a clock offset $t_{0}$ using the $s^{k}$ obtained in the previous step, so as to weaken the effect of clock bias on the TOA measurements in the subsequent solving process. At this point, the objective function is smooth with respect to $t_{0}$. By letting the gradient of $f\left(s^{k},t_{0}\right)$ with respect to $t_{0}$ equal to zero, the closed-form solution of $t_{0}^{k+1}$ can directly obtained as follows:

(10) $$t_{0}^{k+1}=\frac{1}{r+Nc^{2}}\left[c\times \sum_{i=1}^{N}\left(c\times t_{i}-||a_{i}-s^{k}||\right)+rt_{0}^{k}\right].$$

#### 3.1.2. Synchronous Source Localization Subproblem

The clock offset $t_{0}=t_{0}^{k+1}$ estimated in the current subproblem is used to eliminate the distance bias caused by the existence of clock offset in the TOA measurements. With the estimator of the clock offset, the source localization subproblem converts to a synchronous source localization problem, and the source node location estimator $s^{k+1}$ can be expressed as:

(11) $$s^{k+1}=\arg\min_{s}\left\{f\left(s,t_{0}^{k+1}\right)=\frac{1}{2}\sum_{i=1}^{N}{\left[c*\left(t_{i}-t_{0}^{k+1}\right)-||a_{i}-s||\right]}^{2}\right\}.$$

This subproblem estimates the position of source node in an asynchronous system with sensors position and clock offset $t_{0}$. Although we are dealing with an asynchronous source localization problem, when the $t_{0}^{k+1}$ is fixed, the clock offset has been subtracted from the objective function, and the subproblem can be considered as a convex synchronous source localization problem. Then we propose a subgradient descent algorithm for solving it.

Let $\phi_{k}\left(s\right)=f\left(s,t_{0}^{k+1}\right),$ and

(12) $$g^{k}=-\sum_{i=1}^{N}\left[c\times \left(t_{i}-t_{0}^{k+1}\right)-∥a_{i}-s^{k}∥\right]g_{i}^{k},$$

where

(13) $$g_{i}^{k}=\left\{\begin{array}{cc} 0, & \mathrm{if}\;∥a_{i}-s^{k}∥=0,\\ \frac{a_{i}-s^{k}}{∥a_{i}-s^{k}∥}, & \mathrm{otherwise}. \end{array}\right.$$

Then $g_{k}\in \partial \phi_{k}\left(s\right)$ is a subgradient vector of $\phi_{k}\left(s\right)$. In general, the minus subgradient is not a descent direction of a nonsmooth objective function, even if it is convex. However, in $\phi (s,t_{0}^{k+1})$, it has at most one sensor node $a_{i}$ such that $∥a_{i}-s^{k}∥=0$, in which we assign 0 as the subgradient vector of $∥a_{i}-s^{k}∥$. For the other sensor with $∥a_{i}-s^{k}∥\neq 0$, this function is smooth and its gradient can be given by $\frac{a_{i}-s^{k}}{∥a_{i}-s^{k}∥}$. It is easy to verify that, $-g^{k}$ is a descent direction of $\phi (s,t_{0}^{k+1})$ at $s^{k}$. So, we generalize the SABB algorithm proposed by Dong, Li and Peng [16] to the SABB-Subgradient algorithm. By the Barzilai and Borwein method [17], we get a BB step-size as:

(14) $$\alpha_{k}^{BB}=\frac{{\left(v^{k-1}\right)}^{T}v^{k-1}}{{\left(v^{k-1}\right)}^{T}y^{k-1}},$$

where $v^{k-1}=s^{k}-s^{k-1}$ and $y^{k-1}=g^{k}-g^{k-1}$.

We proposed a Barzilai–Borwein subgradient algorithm for the synchronous source localization subproblem as follows Algorithm 1.
**Algorithm 1:** The SABB-Subgradient algorithm for $\min\;\phi_{k}\left(s\right)$**s0.** Let $\left\{\epsilon_{k}\right\}$ be a positive convergent sequence in the sense of $\sum_{k\geq 0}\epsilon_{k}<\infty$, let c∈(0,1), $0<\alpha_{min}<\alpha_{max}<\infty$ and $\alpha_{0}\in \left[\alpha_{min},\alpha_{max}\right]$, $T_{0}>0$, γ∈(0,1), $\vartheta \in Z_{+}$. Set $s^{0}=s^{k}$, l=0.**s1.** Compute $g^{l}$ by (12)–(13). If $∥g^{l}∥<\epsilon_{k}$, then let $s^{k+1}=s^{l}$ and stop.**s2.** Compute ( If l=0, then $\alpha_{l}$ is given by an Armijo line-search.)
(15)$z^{l}=s^{l}-\alpha_{l}g^{l},$ and (16)$\Delta \phi_{k}=\phi_{k}\left(z^{l}\right)-(\phi_{k}\left(s^{l}\right)-c\alpha_{l}∥g^{l}{∥}^{2}).$ Let (17)$p=e^{-\frac{\Delta \phi_{k}}{T_{l}}}.$ Pick a random number (18)$r\in (e^{-\vartheta },e^{-\frac{1}{\vartheta }}).$**s3.** If
(19)p≥r, let $s^{l+1}=z^{l}$ and go to s4. Otherwise, let $\alpha_{l}$ be a step-size given by the Armijo line search, and $s^{l+1}=s^{l}-\alpha_{l}g^{l}$.**s4.** Compute
(20)$$\alpha_{l+1}=\max\left\{\alpha_{min},\min\left\{\alpha_{l}^{BB},\alpha_{max}\right\}\right\},$$ where $\alpha_{l}^{BB}$ is given by (14).**s5.** Let $T_{l+1}:=\gamma T_{l}$, l:=l+1 and go to **s1.**

Combining the solution flow of two subproblems from above, the proximal alternating minimization positioning method for asynchronous TOA-based source localization can be summarized as follows Algorithm 2:
**Algorithm 2:** Proximal Alternating Minimization Positioning (PAMP) method**s0** Initialize: provide a random initial estimator of the location point $s^{0}\in R^{2}$ and initial clock offset $t_{0}^{0}\in R$, respectively. Let ϵ>0 be a small real. Let k:=0.**s1** For the given $\left(s^{k},t_{0}^{k}\right)$, produce $\left(s^{k+1},t_{0}^{k+1}\right)$ via:**s1.1** (Clock Offset Subproblem With known Location) With the source node location $s^{k}$ estimated in the previous step, the $t_{0}$ is estimated via (10);**s1.2** (Synchronous Source Localization Subproblem) Using the estimated $t_{0}$ in the last subproblem to eliminate the influence of clock offset, the asynchronous localization problem is transformed into the synchronous localization problem. Then finding the solution $s^{k+1}$ of this subproblem by Algorithm 1.**s2** If the stopping criterion is met, then stop and let $\left(s^{*},t_{0}^{*}\right):=\left(s^{k+1},t_{0}^{k+1}\right)$ be the final solution; otherwise set k=k+1, go to **s1.**

**Remark** **1.** *In our method, we use $\max\{\left|t_{0}^{k+1}-t_{0}^{k}\right|,||s^{k+1}-s^{k}||\}<\epsilon$ as our stopping criterion, which means the difference between $\left(s^{k+1},t_{0}^{k+1}\right)$ and $\left(s^{k},t_{0}^{k}\right)$ is so subtle that there is no need for further improvement.*

**Figure 2:**
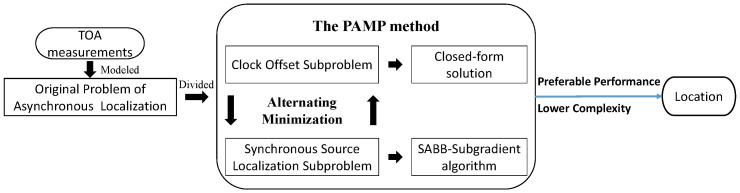
The flowchart for solving asynchronous source localization problem by the proximal alternating minimization positioning (PAMP) method.

Using the PAMP method to solve the asynchronous source localization problem, as shown in Figure 2.

### 3.2. Convergence Analysis

We will prove that sequence $\left\{\left(s^{k},t_{0}^{k}\right)\right\}$ generated by the proposed method is convergent under the Lyapunov framework.

**Definition** **1.** *Let W=X×Y={(u,v)|u∈X,v∈Y}. Function f:W→R is a Lyapunov function associated with a discrete sequence $(u_{k},v_{k})\in W$ if*

*1)*  *f(u,v) is continuous on W;*
*2)*  *The level set {(u,v)|f(u,v)≤c}⊆W is bounded for any positive real c>0;*
*3)*  *$f\left(u_{k+1},v_{k+1}\right)\leq f\left(u_{k},v_{k}\right)$ for all k=1,2,⋯.*

It is easy to verify that, the objective function $f(s,t_{0})$ in problem (7) is continuous on $W=R^{2}\times R$ and, for any c>0 the level set $\left\{\left(s,t_{0}\right)|f\left(s,t_{0}\right)\leq c\right\}$ is bounded. To claim $f(s,t_{0})$ is a Lyapunov function associated with the iteration sequence $\left(s^{k},t_{0}^{k}\right)$ generated by our method, it only needs to prove that $f\left(s^{k+1},t_{0}^{k+1}\right)\leq f\left(s^{k},t_{0}^{k}\right)$.

**Theorem** **1.** *Suppose sequence $\left(s^{k},t_{0}^{k}\right)$ is generated by Algorithm 2, then we have*

(21) $$f\left(s^{k+1},t_{0}^{k+1}\right)\leq f\left(s^{k},t_{0}^{k}\right),\forall k\geq 0.$$

**Proof.**  By iteration (8), $\forall t_{0}\in R$ and $\forall s\in R^{2}$ we have

(22) $$\left\{\begin{array}{cc} f\left(s^{k},t_{0}^{k+1}\right)+\frac{r}{2}{\left(t_{0}^{k+1}-t_{0}^{k}\right)}^{2} & \leq f\left(s^{k},t_{0}\right)+\frac{r}{2}{\left(t_{0}-t_{0}^{k}\right)}^{2},\\ f(s^{k+1},t_{0}^{k+1}) & \leq f(s,t_{0}^{k+1}). \end{array}\right.$$

Set $\left(s,t_{0}\right)=\left(s^{k},t_{0}^{k}\right)$, we get

(23) $$\left\{\begin{array}{cc} f\left(s^{k},t_{0}^{k+1}\right)+\frac{r}{2}{\left(t_{0}^{k+1}-t_{0}^{k}\right)}^{2} & \leq f(s^{k},t_{0}^{k}),\\ f(s^{k+1},t_{0}^{k+1}) & \leq f(s^{k},t_{0}^{k+1}). \end{array}\right.$$

Adding two inequalities in (23) we obtain

(24) $$f\left(s^{k+1},t_{0}^{k+1}\right)\leq f\left(s^{k},t_{0}^{k}\right)-\frac{r}{2}{\left(t_{0}^{k+1}-t_{0}^{k}\right)}^{2}\leq f\left(s^{k},t_{0}^{k}\right).$$

In the last inequality, equality holds if and only if $t_{0}^{k+1}=t_{0}^{k}$.

By LaSalle invariance principle [18], we immediately have

**Theorem** **2.** *The sequence $\left(s^{k},t_{0}^{k}\right)$ generated by our Proximal Alternating Minimization Positioning Method (Algorithm 2) converges to Lyapunov stationary point of problem (7).* □

## 4. Performance and Complexity Analysis

### 4.1. Cramér–Rao Lower Bound

By the asynchronous TOA-Based measurement model (7), the performance of any unbiased estimation of *s* is limited by the Cramér–Rao Lower Bound (CRLB) [19]. In order to obtain the CRLB under the asynchronous TOA-Based measurement model, we assume that the measurement noises in (6) are independent and identically distributed (i.i.d) Gaussian random variables with zero mean and variance $\sigma^{2}$. Under this assumption, the joint conditional probability density function of the measured data $t_{i}$ is:

(25) $$\begin{array}{cc} p\left(t_{1},t_{2},\ldots ,t_{N}|s,t_{0}\right) & =\prod_{i=1}^{N}\frac{1}{\sqrt{2\pi \sigma_{i}^{2}}}\times \exp\left(-\frac{1}{2\sigma^{2}}{\left(c*\left(t_{i}-t_{0}\right)-{\left||a_{i}-s|\right|}_{2}\right)}^{2}\right)\\ \; & =\frac{1}{{\left(2\pi \sigma_{i}^{2}\right)}^{\frac{N}{2}}}\exp\left(-\frac{1}{2\sigma^{2}}\sum_{i=1}^{N}{\left(c*\left(t_{i}-t_{0}\right)-{\left||a_{i}-s|\right|}_{2}\right)}^{2}\right). \end{array}$$

Let $a_{ij},s_{j}$ denote the $j^{th}$ element of $i^{th}$ sensor node $a_{i}$ and source node *s* respectively. Let $\varphi ={\left[s_{1},\ldots ,s_{m},t_{0}\right]}^{T}$ be a vector consisting of all unknowns, where m=2. The log-likelihood function (ignoring the constant term) is written as:

(26) $$L\left(\varphi \right)=-\frac{1}{2\sigma^{2}}\sum_{i=1}^{N}{\left(c*\left(t_{i}-t_{0}\right)-{\left||a_{i}-s|\right|}_{2}\right)}^{2}.$$

Each element of Fisher information matrix FIM(φ) is given by: for 1≤j≤k≤m,

(27) $$\begin{array}{cc} {\left[\mathrm{FIM}(\varphi )\right]}_{jk} & =-E\left(\frac{\partial^{2}}{\partial \varphi_{j}\partial \varphi_{k}}L\left(\varphi \right)\right)\\ \; & =\frac{1}{\sigma^{2}}\sum_{i=1}^{N}\frac{\left(a_{ij}-s_{j}\right)\left(a_{ik}-s_{k}\right)}{{\left||a_{i}-s|\right|}^{2}} \end{array}$$

and for 1≤j≤m,

(28) $$\begin{array}{cc} {\left[\mathrm{FIM}(\varphi )\right]}_{j\left(m+1\right)} & ={\left[\mathrm{FIM}(\varphi )\right]}_{\left(m+1\right)j}\\ & =-E\left(\frac{\partial^{2}}{\partial \varphi_{j}\partial \varphi_{m+1}}\ln p\left(t_{1},\ldots ,t_{N}|\varphi \right)\right)\\ \; & =\frac{1}{\sigma^{2}}\sum_{i=1}^{N}\frac{a_{ij}-s_{j}}{\left||a_{i}-s|\right|} \end{array}$$

and

(29) $${\left[\mathrm{FIM}(\varphi )\right]}_{\left(m+1\right)\left(m+1\right)}=-E\left(\frac{\partial^{2}}{\partial \varphi_{m+1}\partial \varphi_{m+1}}L\left(\varphi \right)\right)=\frac{N}{\sigma^{2}}.$$

So the FIM is:

(30) $$\begin{array}{c} \left[\mathrm{FIM}(\varphi )\right]=\frac{1}{\sigma^{2}}\times \left(\begin{array}{ccc} \sum_{i=1}^{N}\frac{\left(a_{i1}-s_{1}\right)\left(a_{i1}-s_{1}\right)}{{\left||a_{i}-s|\right|}^{2}} & \sum_{i=1}^{N}\frac{\left(a_{i1}-s_{1}\right)\left(a_{i2}-s_{2}\right)}{{\left||a_{i}-s|\right|}^{2}} & \sum_{i=1}^{N}\frac{a_{i1}-s_{1}}{\left||a_{i}-s|\right|}\\ \sum_{i=1}^{N}\frac{\left(a_{i2}-s_{2}\right)\left(a_{i1}-s_{1}\right)}{{\left||a_{i}-s|\right|}^{2}} & \sum_{i=1}^{N}\frac{\left(a_{i2}-s_{2}\right)\left(a_{i2}-s_{2}\right)}{{\left||a_{i}-s|\right|}^{2}} & \sum_{i=1}^{N}\frac{a_{i2}-s_{2}}{\left||a_{i}-s|\right|}\\ \sum_{i=1}^{N}\frac{a_{i1}-s_{1}}{\left||a_{i}-s|\right|} & \sum_{i=1}^{N}\frac{a_{i2}-s_{2}}{\left||a_{i}-s|\right|} & \frac{N}{\sigma^{2}} \end{array}\right). \end{array}$$

The diagonal element of the inverse FIM is the minimum variance that can be reached theoretically. Therefore, the CRLB of unbiased estimation $\hat{s}$ is:

(31) $${\mathrm{MSE}}_{\varphi }=E\left({\left||\hat{s}-s|\right|}^{2}\right)\geq \sum_{i=1}^{m}{\left[\mathrm{FIM}{\left(\varphi \right)}^{-1}\right]}_{ii},$$

and

(32) $$\begin{array}{c} {\mathrm{RMSE}}_{\varphi }=\sqrt{\frac{1}{M}{\sum_{p=1}^{M}\left({\mathrm{MSE}}_{\varphi }\right)}_{p}}\geq \sqrt{\frac{1}{M}{\sum_{p=1}^{M}\left(\sum_{i=1}^{m}{\left[\mathrm{FIM}{\left(\varphi \right)}^{-1}\right]}_{ii}\right)}_{p}}, \end{array}$$

where *M* is the number of Monte Carlo simulations.

### 4.2. Simulation Results

#### 4.2.1. Simulation Settings

The numerical performance of the proposed method is examined in two scenarios: the deterministic sensor location (scenario 1) and uniformly distributed sensor location (scenario 2).

In scenario 1, both near-field and far-field cases are investigated. By near-field/far-field, we mean that the source node is inside/outside the convex hull formed by sensors. To be specific, in scenario 1, we consider the same sensor deployment used as in [12]. There are eight sensors with positions: $a_{1}={\left(400,400\right)}^{T}$, $a_{2}={\left(400,-400\right)}^{T}$, $a_{3}={\left(-400,400\right)}^{T}$, $a_{4}={\left(-400,-400\right)}^{T}$, $a_{5}={\left(800,800\right)}^{T}$, $a_{6}={\left(800,-800\right)}^{T}$, $a_{7}={\left(-800,800\right)}^{T}$ and $a_{8}={\left(-800,-800\right)}^{T}\left(\mathrm{unit}:\;m\right)$. The true position of the source node in the case of the near-field is $s={\left(30,10\right)}^{T}$ and the far-field is $s={\left(1350,10\right)}^{T}$, respectively.

In scenario 2, the positions of the eight sensors are the same as in scenario 1, the source node is uniformly distributed in a square region of size $\left[-1200m,1200m\right]\times \left[-1200m,1200m\right]$. In both scenarios 1 and 2, $t_{0}$ follows a Gaussian distribution $N\left(0,16\right)ns$. What needs to be explained here is that our algorithm does not depend on specific noise distribution and does not need to use noise structure in the process of solving. In order to compare with the CRLB and existing algorithms, the noise is set as two distributions in following simulation (i.e., Gaussian noise and uniformly distributed noise).

The performance of the proposed method is compared with the 2LS and MMA algorithms in [12], the SDP-NEW approach in [13] and the SDP2016 approach in [14]. The source codes of the PAMP method is available at [20].The localization accuracy is evaluated in terms of the root-mean square error (RMSE), it is

(33) $$\mathrm{RMSE}=\sqrt{\frac{1}{M}\sum_{i=1}^{M}∥\hat{s_{i}}{-s∥}^{2}},$$

where *s* is the true location of source node, ${\hat{s}}_{i}$ is the estimated location of source node at ith Monte Carlo simulation, M=1000 which is the number of simulations.

The simulation scenario considered in [14] is small, and the choice of penalty factor is not comprehensive enough. The simulation scenario in this paper is based on the actual engineering problems, so the considerations are more comprehensive. To be fair, we take *K* constant value $\eta_{k}$, k=1,2,…,K to compute the cost function of SDP2016 algorithm, and then the $J_{k}$ function given by [14] is used to select the optimal penalty factor for our simulation. For the 2LS approach, the penalty factor is set to $6.18\times 10^{-5}$. In our simulations, the noise in the time domain $n_{i}$ in (6) is transformed into the distance domain similar to [12], and it is set to be the same for simplicity of illustration.

#### 4.2.2. Performance Comparisons

**Figure 3:**
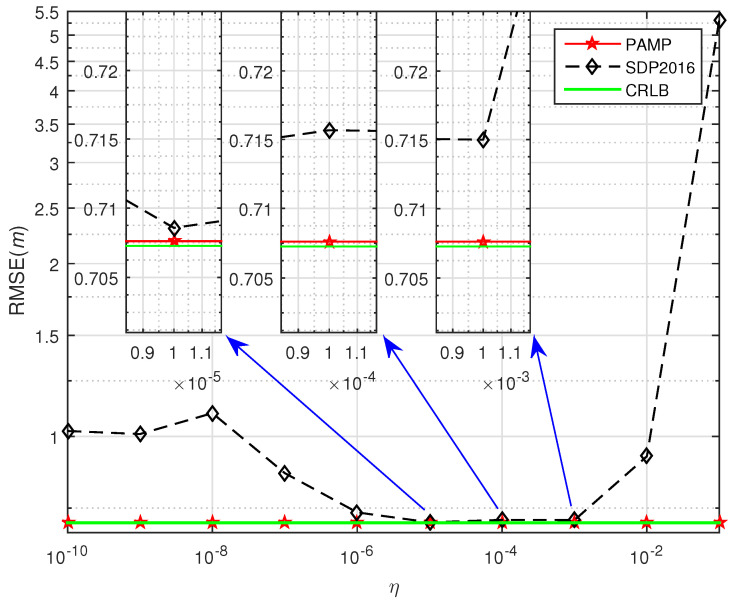
Selection of the penalty factor η in SDP2016 when $\sigma^{2}$ = 0 dB for near-field case in scenario 1.

**Figure 4:**
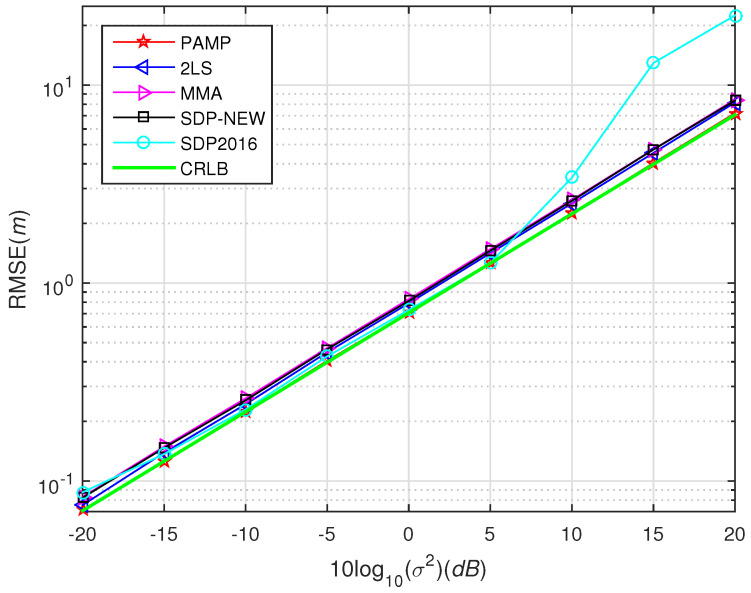
Root-mean square error (RMSE) versus $\sigma^{2}$ using different methods, near-field case in scenario 1.

**Figure 5:**
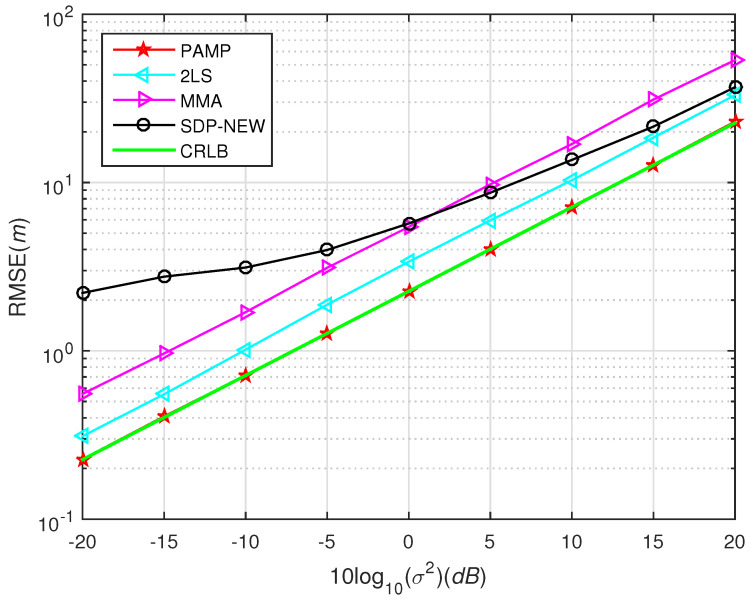
RMSE versus $\sigma^{2}$ using different methods, far-field case in scenario 1.

**Figure 6:**
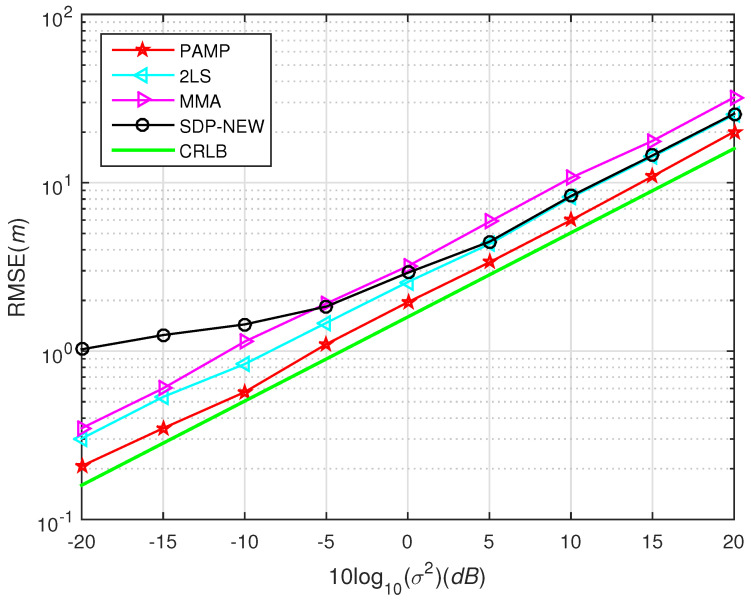
RMSE versus $\sigma^{2}$ using different methods in scenario 2, Gaussian noise.

**Figure 7:**
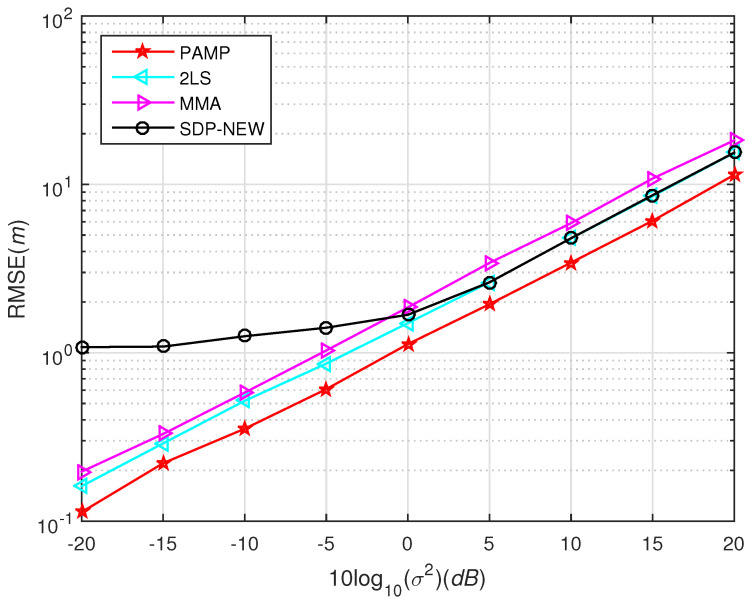
RMSE versus $\sigma^{2}$ using different methods in scenario 2, uniformly distributed noise.

In Figure 1, we test the performance of SDP2016 approach at different η and compared it with the proposed method. The performance of the proposed method under different $\sigma^{2}$ with compared to some existing algorithms in scenario 1 and scenario 2 are shown from Figure 3, Figure 4, Figure 5, Figure 6 and Figure 7, respectively. For comparisons, the root CRLB is also displayed. It is observed that, the proposed method (PAMP) outperforms 2LS, MMA, SDP-NEW and SDP2016 algorithms in all cases.

Simulation 1:

Due to an increase in the area considered for simulation, the original penalty factors of SDP2016 algorithm cannot be directly used in this simulation. We selected the appropriate penalty factor by testing the sensitivity of the penalty factor in SDP2016 approach. We fix the $\sigma^{2}$ to 0 dB and the source position to ${\left(30,10\right)}^{T}$. Figure 3 compares RMSE with different $\sigma^{2}$. It can be seen that the performance of SDP2016 algorithm is sensitive to the choice of penalty factor η. Similarly, for different $\sigma^{2}$ in the same scenario, the SDP2016 algorithm needs to use different penalty factors to improve the positioning accuracy. When $\sigma^{2}$ = 0 dB for near-field case in scenario 1, SDP2016 algorithm achieves the best performance with RMSE =0.709m when the penalty factor $\eta =1.0\times 10^{-5}$, and $\eta =1.0\times 10^{-4}$ it obtains RMSE >0.715m and $\eta =1.0\times 10^{-3}$ it gets 0.710m< RMSE ≤0.715m, but our method always achieves RMSE <0.709m. After this test, the penalty factor of SDP2016 is set to vary from $10^{-10}$ to $10^{-2}$. Obviously, the PAMP method does not contain penalty factor, it can accurately and stably estimate the location of source node in any scenario.

Simulation 2: 

The source is placed at point $s={\left(30,10\right)}^{T}$, which is inside the convex hull formed by the sensor nodes. The noise is generated from i.i.d. Gaussian, and $t_{0}$ is randomly chosen by normal distribution with zero mean and variance $4^{2}$. In Figure 4, we compare the performance of our PAMP and 2LS, MMA, SDP-NEW and SDP2016 algorithms. It can be found that our PAMP method consistently outperforms the other algorithms in terms of the gap between the solution and CRLB. The PAMP method is closest to the CRLB, followed by SDP2016, 2LS, SDP-NEW and MMA. It is worth noting that the performance of SDP2016 algorithm is poor in high-noise environment, but when $\sigma^{2}$ decreases to 5 dB, it improves to be second only to that of PAMP method. However, the PAMP method still has incomparable advantages over other algorithms, because it does not require noise information.

In order to compare performance conveniently, the following Table 1 lists the RMSE comparison between the PAMP and the SDP2016 under different $\sigma^{2}$ and the optimal penalty factors required by SDP2016 algorithm in each cases. This experiment further demonstrates that SDP2016 algorithm is sensitive to penalty factors, and it is difficult to select an appropriate penalty factor in different scenarios, which consumes too much work. Based on the above considerations, the subsequent simulation will not be compared with the SDP2016 algorithm.

Simulation 3: 

We locate the source node at $s={\left(1350,10\right)}^{T}$, which is outside the convex hull of the sensor nodes. The noise follows from the normally distributed with zero mean and variance $4^{2}$ and i.i.d. Gaussian measurement noise. In Figure 5, unfortunately, SDP-NEW approach does not give a good estimation when $\sigma^{2}$ decreases to 5 dB in this case. One reason is that the source node is not in the convex hull and SDP optimization cannot find a better solution. It can find that PAMP method consistently provides the best performance. In other words, the PAMP method has excellent anti-noise performance in high noise environment.

Simulation 4: 

The source node is uniformly distributed in a square region [−1200 m, 1200 m] × [−1200 m, 1200 m]. For each $\sigma^{2}$, we randomly generate 1000 source locations. The noise is i.i.d. Gaussian and $t_{0}$ is randomly chosen from the normal distribution with zero mean and variance $4^{2}$. We display the performance of different algorithms in Figure 6. As it is in the previous simulation, the PAMP method provides excellent performance in a high noise environment.

Simulation 5: 

The source node is uniformly distributed in a square region [−1200 m, 1200 m] × [−1200 m, 1200 m]. Different from Simulation 4, the environment noise is set as uniformly distributed in this simulation and $t_{0}$ is randomly chosen from the normal distribution with zero mean and variance $4^{2}$. Since the CRLB is derived under the condition of Gaussian noise, the CRLB is not shown in Figure 7 for uniformly distributed noise. From Figure 7 we can find that the positioning accuracy of all algorithms is improved when the noise is uniformly distributed. The proposed PAMP method is still far ahead in performance comparison.

#### 4.2.3. Complexity Comparisons

In scenario 1 and scenario 2 when $\sigma^{2}$ = 0 dB, the computational time and iteration number of proposed PAMP method and other algorithms are shown from Table 2, Table 3, Table 4 and Table 5 for comparison. In Table 6, we summarize algorithm complexity in terms of operations in each iteration. The code is run on a personal computer with Intel(R) Core(TM) i5-4590 CPU 3.30GHz and 8 GB RAM.

It is obvious that, the PAMP method is superior to the other algorithms [12,13,14] in terms of computational time. This is guaranteed by the nature of proximal alternating minimization and excellent performance of SABB-Subgradient algorithm. Each step of the PAMP method contains an closed-form solution and subproblem iteration, where the subproblem is solved by SABB-Subgradient algorithm. This approach is an efficient first-order acceleration algorithm which has been verified by a large number of numerical experiments.

Suppose *m* is the dimension of the problem, *N* is the number of sensors and $\bar{t}$ is the average number of subproblem iterations, which is usually less than 5. The overhead of subproblem is $O(\left(mN\right)\bar{t})$, and then consider the cost of closed-form solution, we can get the operation per iteration of the PAMP method is only $O(\left(mN\right)\bar{t}+1)$. Obviously $\left(mN\right)\bar{t}+1$ is a linear function of the problem size *m*, which means that the cost of one-step iteration of the PAMP method is negligible. The algorithms we compared use SDP technique to solve this sticky problem, which ascends the dimension of the original problem from *m* to *l*, where $l=(N^{2}+N+m+1)$. In the SeDuMi solver, the cost of each step is at least Poly(l(l+1)+c), see Roos, Terlaky and Vial [21].

For this reason, even though the PAMP method has more iterations in scenarios 1 and 2 when the source node is outside the convex hull. Yet the subproblem is quickly solved by SABB-Subgradient algorithm, hence the computational time is still less than other algorithms at least by one order of magnitude.

#### 4.2.4. Summary

One can find the proposed algorithm provides better estimation and faster convergence than traditional SDP approaches. The 2LS and MMA algorithms adopt different objective functions, the former includes extra penalty to improve the tightness of cost function, so it can achieve higher positioning accuracy, but also correspondingly improve the computation time and iteration number. SDP-NEW algorithm ranked fourth in performance comparison of scenario 1 near field. Once the source is outside the convex hull, the localization performance decreases rapidly, and the robustness is not as good as MMA algorithm. Although an optimal penalty factor can improve the performance of SDP2016 algorithm, which is second only to PAMP method, the selection of penalty factor is relatively complex and it is difficult to be applied in practical applications. In a word, our approach will more likely to find the approximating global solution in complex engineering environment at higher speed.

The objective function $f(s,t_{0})$ in (7) is non-convex and non-smooth for the jointed variable $\left(s,t_{0}\right)$. It is hard to find the global minimizer of problem (7). By convex relaxation, some convex optimization algorithms can be used for the approximate convex problem [22]. The convex relaxation approach has suboptimal solution with high computational complexity. By smoothing objective function $f(s,t_{0})$, the trust region algorithm can be used for problem (7) [23]. These methods mentioned above require relaxation or approximation of the objective function, which may result in some performance loss of TOA. In this paper, we propose a proximal alternating minimization positioning method for problem (7). The proposed method optimizes the original objective function in an alternative mode. At each iteration, the biconvexity of the objective function yields two easy subproblems: the clock offset subproblem has a closed-form solution, and the synchronous source localization subproblem is a convex optimization problem which is easy to obtain an approximating global solution.

## 5. Conclusions

In this paper, we investigated the TOA-based source localization with unknown clock offset via a biconvex minimization model, and proposed a proximal alternating minimization positioning method to solve the original model. We also proved the global convergence of proposed method under the Lyapunov framework. Simulation results show that the performance of PAMP method in the problem of asynchronous TOA-based source localization is closest to the CRLB. When the target is randomly distributed around the sensors, the operation time for precise positioning is at least one order of magnitude less than the classic algorithm when using the PAMP method.

## Figures and Tables

**Table 1:** RMSE comparison (in meter) and penalty factor selection under different $\sigma^{2}$ for the near-field case in scenario 1.

$\sigma^{2}$	RMSE of PAMP	RMSE of SDP2016 (Optimal η)
−20 dB	0.072	0.088 ($10^{-7}$)
−15 dB	0.126	0.137 ($10^{-7}$)
−10 dB	0.224	0.230 ($10^{-7}$)
−5 dB	0.405	0.426 ($10^{-4}$)
0 dB	0.711	0.731 ($10^{-5}$)
5 dB	1.262	1.264 ($10^{-9}$)
10 dB	2.243	3.406 ($10^{-8}$)
15 dB	4.037	12.930 ($10^{-8}$)
20 dB	7.204	22.477 ($10^{-4}$)

**Table 2:** Average computational time (in seconds) and average iteration number for the near-field case in scenario 1.

Method	Computational Time	Iteration Number
Proposed PAMP	$1.30\times 10^{-3}$	3.83
2LS in [12]	$5.71\times 10^{-1}$	37.29
MMA in [12]	$3.53\times 10^{-1}$	11.04
SDP-NEW in [13]	$3.29\times 10^{-1}$	23.34
SDP2016 in [14]	$9.87\times 10^{-1}$	32.96

**Table 3:** Average computational time (in seconds) and average iteration number for the far-field case in scenario 1.

Method	Computational Time	Iteration Number
Proposed PAMP	$9.13\times 10^{-2}$	454.10
2LS in [12]	$5.76\times 10^{-1}$	37.31
MMA in [12]	$3.57\times 10^{-1}$	12.38
SDP-NEW in [13]	$3.30\times 10^{-1}$	23.99

**Table 4:** Average computational time (in seconds) and average iteration number in scenario 2, Gaussian noise.

Method	Computational Time	Iteration Number
Proposed PAMP	$6.66\times 10^{-2}$	219.60
2LS in [12]	$6.11\times 10^{-1}$	37.73
MMA in [12]	$3.82\times 10^{-1}$	11.94
SDP-NEW in [13]	$3.64\times 10^{-1}$	24.65

**Table 5:** Average computational time (in seconds) and average iteration number in scenario 2, uniformly distributed noise.

Method	Computational Time	Iteration Number
Proposed PAMP	$7.82\times 10^{-2}$	268.41
2LS in [12]	$5.63\times 10^{-1}$	37.70
MMA in [12]	$3.17\times 10^{-1}$	11.71
SDP-NEW in [13]	$3.35\times 10^{-1}$	24.62

**Table 6:** Complexity comparison.

Method	Operation Per Iteration
Proposed PAMP	$O(\left(mN\right)\bar{t}+1)$
2LS, MMA, SDP-NEW and SDP2016	Poly(l(l+1)+c)

## References

[1] Akyildiz I.F., Su W., Sankarasubramaniam Y., Cayirci E. (2002). Wireless sensor networks: A survey. Comput. Netw..

[2] Sayed A.H., Tarighat A., Khajehnouri N. (2005). Network-based wireless location: Challenges faced in developing techniques for accurate wireless location information. IEEE Signal Process. Mag..

[3] Zekavat R., Buehrer R.M. (2019). Source Localization: Algorithms and Analysis. Handbook of Position Location: Theory, Practice, and Advances.

[4] Vaghefi R.M., Gholami M.R., Buehrer R.M., Strom E.G. (2012). Cooperative received signal strength-based sensor localization with unknown transmit powers. IEEE Trans. Signal Process..

[5] Gavish M., Weiss A.J. (1992). Performance analysis of bearing-only target location algorithms. IEEE Trans. Aerosp. Electron. Syst..

[6] Cheung K.W., So H.C., Ma W.K., Chan Y.T. (2004). Least squares algorithms for time-of-arrival-based mobile location. IEEE Trans. Signal Process..

[7] Yang K., Wang G., Luo Z.Q. (2009). Efficient convex relaxation methods for robust target localization by a sensor network using time differences of arrivals. IEEE Trans. Signal Process..

[8] Taponecco L., D’Amico A.A., Mengali U. (2011). Joint TOA and AOA estimation for UWB localization applications. IEEE Trans. Wirel. Commun..

[9] Erol-Kantarci M., Mouftah H.T., Oktug S. (2011). A survey of architectures and localization techniques for underwater acoustic sensor networks. IEEE Commun. Surv. Tutor..

[10] McCrady D.D., Doyle L., Forstrom H., Dempsey T., Martorana M. (2000). Mobile ranging using low-accuracy clocks. IEEE Trans. Microw. Theory Technol..

[11] Zheng J., Wu Y.C. (2009). Joint time synchronization and localization of an unknown node in wireless sensor networks. IEEE Trans. Signal Process..

[12] Xu E., Ding Z., Dasgupta S. (2011). Source localization in wireless sensor networks from signal time-of-arrival measurements. IEEE Trans. Signal Process..

[13] Vaghefi R.M., Buehrer R.M. Asynchronous time-of-arrival-based source localization. Proceedings of the 2013 IEEE International Conference on Acoustics, Speech and Signal Processing.

[14] Zou Y., Wan Q. (2016). Asynchronous time-of-arrival-based source localization with sensor position uncertainties. IEEE Commun. Lett..

[15] Beck A., Stoica P., Li J. (2008). Exact and approximate solutions of source localization problems. IEEE Trans. Signal Process..

[16] Dong W.L., Li X., Peng Z. (2019). A Simulated Annealing-Based Barzilai–Borwein Gradient Method for Unconstrained Optimization Problems. Asia Pac. J. Oper. Res..

[17] Barzilai J., Borwein J.M. (1988). Two-point step size gradient methods. IMA J. Numer. Anal..

[18] LaSalle J.P. (1976). The Stability of Dynamical Systems.

[19] Kay S.M. (1993). Fundamentals of Statistical Signal Processing.

[20] Chen Y., Yao Z., Peng Z. The PAMP Method. https://github.com/LeslieChan0513/localization-method.

[21] Roos C., Terlaky T., Vial J.P. (2005). A Polynomial Algorithm for the Self-dual Model. Interior Point Methods for Linear Optimization.

[22] Boyd S., Vandenberghe L. (2004). Convex Optimization.

[23] Yao Z., Huang J., Wang S., Ruby R. (2017). Efficient local optimisation-based approach for non-convex and non-smooth source localisation problems. IET Radar Sonar Navig..

